# Supplementary material for: The L467F-F508del Complex Allele Hampers Pharmacological Rescue of Mutant CFTR by Elexacaftor/Tezacaftor/Ivacaftor in Cystic Fibrosis Patients: The Value of the Ex Vivo Nasal Epithelial Model to Address Non-Responders to CFTR-Modulating Drugs
Source: Int J Mol Sci. 2022 Mar 15;23(6):3175. doi: 10.3390/ijms23063175 (PMC8952007; doi:10.3390/ijms23063175)
Supplement: Supplementary file 1 [file ijms-23-03175-s001.zip › Suppl Figure S1.pdf]

International Journal of *Molecular Sciences*

MDPI

Supplementary Figure

The L467F-F508del Complex Allele Hampers Pharmacological Rescue of Mutant CFTR by Elexacaftor/Tezacaftor/Ivacaftor in Cystic Fibrosis Patients: The Value of the Ex Vivo Nasal Epithelial Model to Address Non-Responders to CFTR-Modulating Drugs

Elvira Sondo [1,†], Federico Cresta [2,†], Cristina Pastorino [3], Valeria Tomati [1], Valeria Capurro [1], Emanuela Pesce [1], Mariateresa Lena [1], Michele Iacomino [1], Ave Maria Baffico [4], Domenico Coviello [4], Tiziano Bandiera [5], Federico Zara [1,3], Luis J. V. Galietta [6,7], Renata Bocciardi [1,3], Carlo Castellani [2,‡,*] and Nicoletta Pedemonte [1,‡,*]

1 UOC Genetica Medica, IRCCS Istituto Giannina Gaslini, 16147 Genova, Italy; elvirasondo@gaslini.org (E.S.); valeriatomati@gaslini.org (V.T.); valeriacapurro@gaslini.org (V.C.); emanuela.pesce@yahoo.it (E.P.); mariateresalena@gaslini.org (M.L.); micheleiacomino@gaslini.org (M.I.); federico.zara@unige.it (F.Z.); renata.bocciardi@unige.it (R.B.)
2 UOSD Centro Fibrosi Cistica, IRCCS Istituto Giannina Gaslini, 16147 Genova, Italy; federicocresta@gaslini.org
3 Department of Neurosciences, Rehabilitation, Ophthalmology, Genetics, Maternal and Child Health (DINOGMI), University of Genoa, 16132 Genova, Italy; cristinapastorino22@gmail.com
4 UOC Laboratorio di Genetica Umana, IRCCS Istituto Giannina Gaslini, 16147 Genova, Italy; avemariabaffico@gaslini.org (A.M.B.); domenicocoviello@gaslini.org (D.C.)
5 D3-PharmaChemistry, Fondazione Istituto Italiano di Tecnologia, 16163 Genova, Italy; tiziano.bandiera@iit.it
6 Telethon Institute of Genetics and Medicine (TIGEM), 80078 Pozzuoli, Italy; l.galietta@tigem.it
7 Department of Translational Medical Sciences (DISMET), University of Naples Federico II, 80131 Naples, Italy
* Correspondence: carlocastellani@gaslini.org (C.C.); nicolettapedemonte@gaslini.org (N.P.)
† These authors contributed equally as first authors.
‡ These authors contributed equally as last authors.

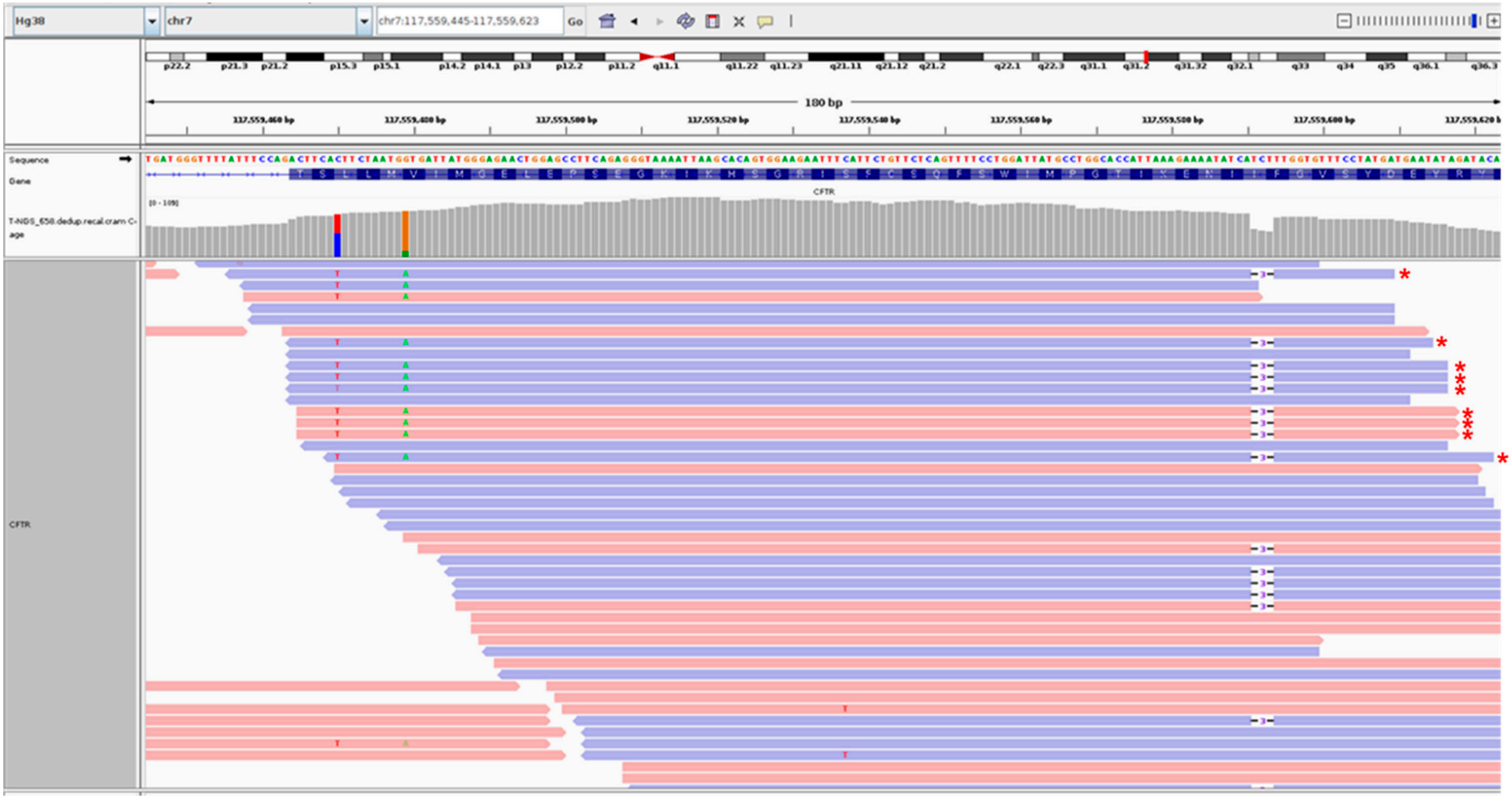

Figure S1. The L467/F508del complex allele and the Gaslini exome genomic database. Genomic window spanning a region of the *CFTR* exon 11 generated by the Integrative Genomics Viewer (IGV) and displaying the BAM alignment track mapped read-segments per reference sequence (GRCh38/hg38). Red asterisks indicate the reads showing the mismatch positions corresponding to the presence of the L467F (T, red) and F508del (-3-) variants in *cis* in the same sequence fragment. A further mismatch (A, green) in cis with the two variants corresponds to the presence of the V470M polymorphism
