# Supplementary material for: The L467F-F508del Complex Allele Hampers Pharmacological Rescue of Mutant CFTR by Elexacaftor/Tezacaftor/Ivacaftor in Cystic Fibrosis Patients: The Value of the Ex Vivo Nasal Epithelial Model to Address Non-Responders to CFTR-Modulating Drugs
Source: Int J Mol Sci. 2022 Mar 15;23(6):3175. doi: 10.3390/ijms23063175 (PMC8952007; doi:10.3390/ijms23063175)
Supplement: Supplementary file 1 [file ijms-23-03175-s001.zip › Supplemental File S1 - final.pdf]

International Journal of *Molecular Sciences*

MDPI

Supplementary File

The L467F-F508del Complex Allele Hampers Pharmacological Rescue of Mutant CFTR by Elexacaftor/Tezacaftor/Ivacaftor in Cystic Fibrosis Patients: The Value of the Ex Vivo Nasal Epithelial Model to Address Non-Responders to CFTR-Modulating Drugs

Elvira Sondo [1,†], Federico Cresta [2,†], Cristina Pastorino [3], Valeria Tomati [1], Valeria Capurro [1], Emanuela Pesce [1], Mariateresa Lena [1], Michele Iacomino [1], Ave Maria Baffico [4], Domenico Coviello [4], Tiziano Bandiera [5], Federico Zara [1,3], Luis J. V. Galietta [6,7], Renata Bocciardi [1,3], Carlo Castellani [2,‡,*] and Nicoletta Pedemonte [1,‡,*]

[1] UOC Genetica Medica, IRCCS Istituto Giannina Gaslini, 16147 Genova, Italy; elvirasondo@gaslini.org (E.S.); valeriatomati@gaslini.org (V.T.); valeriacapurro@gaslini.org (V.C.); emanuela.pesce@yahoo.it (E.P.); mariateresalena@gaslini.org (M.L.); micheleiacomino@gaslini.org (M.I.); federico.zara@unige.it (F.Z.); renata.bocciardi@unige.it (R.B.)

[2] UOSD Centro Fibrosi Cistica, IRCCS Istituto Giannina Gaslini, 16147 Genova, Italy; federicocresta@gaslini.org

[3] Department of Neurosciences, Rehabilitation, Ophthalmology, Genetics, Maternal and Child Health (DINOGMI), University of Genoa, 16132 Genova, Italy; cristinapastorino22@gmail.com

[4] UOC Laboratorio di Genetica Umana, IRCCS Istituto Giannina Gaslini, 16147 Genova, Italy; avemariabaffico@gaslini.org (A.M.B.); domenicocoviello@gaslini.org (D.C.)

[5] D3-PharmaChemistry, Fondazione Istituto Italiano di Tecnologia, 16163 Genova, Italy; tiziano.bandiera@iit.it

[6] Telethon Institute of Genetics and Medicine (TIGEM), 80078 Pozzuoli, Italy; l.galietta@tigem.it

[7] Department of Translational Medical Sciences (DISMET), University of Naples Federico II, 80131 Naples, Italy

* Correspondence: carlocastellani@gaslini.org (C.C.); nicolettapedemonte@gaslini.org (N.P.)

† These authors contributed equally as first authors.

‡ These authors contributed equally as last authors.

TT001–clinical data

TT001 is a 33-year-old male diagnosed with CF at one year of age. His sweat chloride was 130 mEq/L and a CFTR mutation panel identified F508del and G542X. His history of CF is characterized by pancreatic insufficiency, severe lung disease, pulmonary colonization by Methicillin-Sensitive *Staphylococcus aureus* (MSSA) and *Exophiala dermatitidis*. Attempts to eradicate *E. dermatitidis* with posaconazole and isavuconazole proved unsuccessful. Since 2017 he has yearly hospital admissions for pulmonary exacerbations (PEx), and in 2018 has started non-invasive ventilation during night rest and exercise. His ppFEV1 is stably under 40%, even after IV antibiotics.

In January 2020 he was started on Elexacaftor/Tezacaftor/Ivacaftor (ETI). ETI was well tolerated, and within 2 days of treatment ppFEV1 moved from 36% to 51% and the patient initially reported less cough and dyspnea. However, in the following months the cough increased again, and in spite of continuative oral antibiotics ppFEV1 decreased until pre-ETI levels [Table S1]. Further post-ETI sweat chloride levels showed a modest decrease, never reaching values below 100 mEq/L [Table S1].

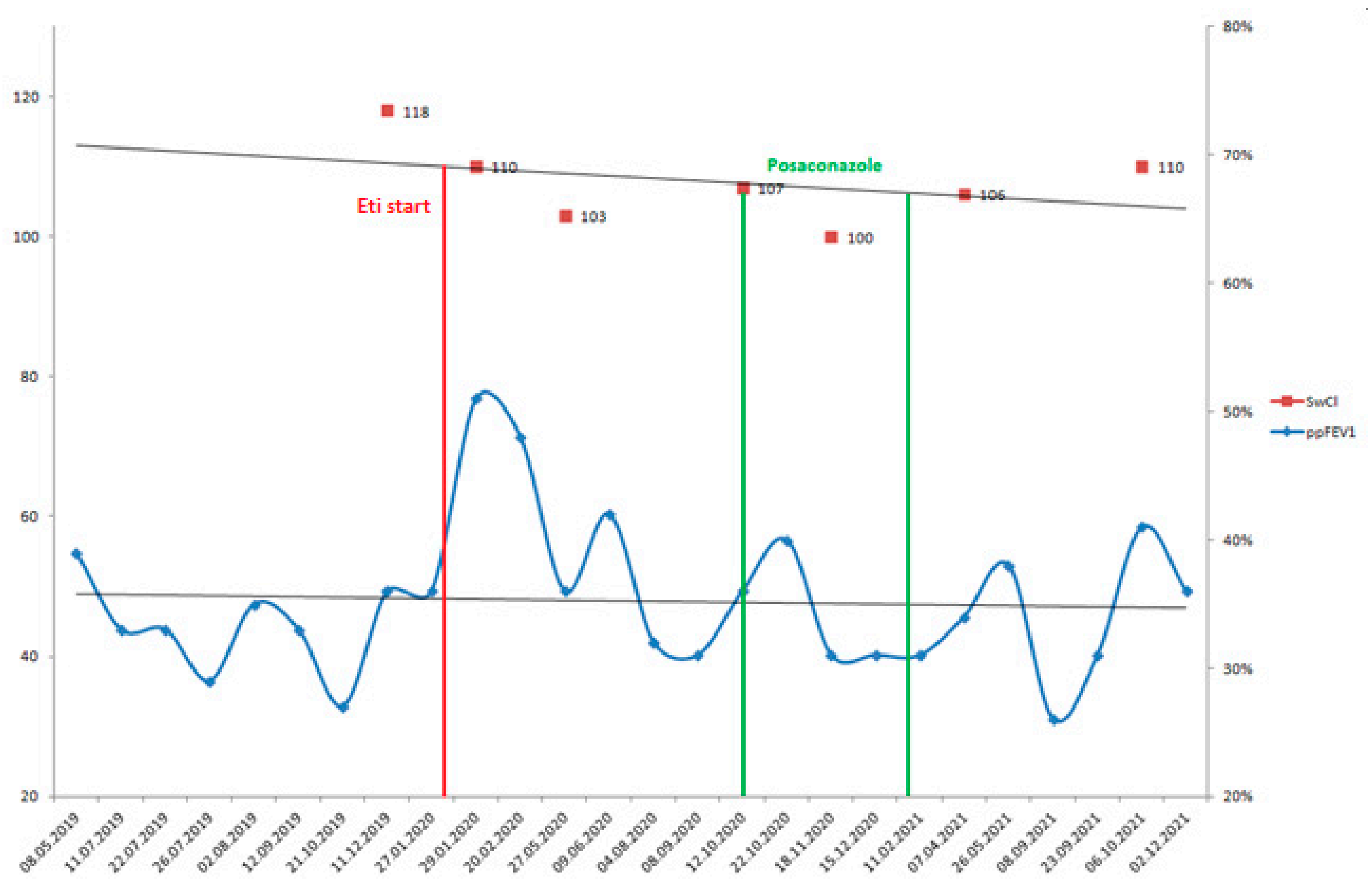

Table S1

In consideration of the disappointing response to modulators and following another sputum culture positive for *E. dermatitidis,* we started posaconazole (300 mg SID). Azoles are strong inhibitors of Cytp450, an enzyme metabolizin CFTR modulators, and ETI dose adjustment is needed when the two medicines are taken together. Under these circumstances, the drug brochure recommends stopping the Ivacaftor evening dose and administer the ETI morning dose initially following a 1 day on 2 days off schedule and then twice a week. We chose a different approach and, after obtaining the patient's consent, began the standard dosage on alternate days.

This was done because, in order to explain the absence of sustained clinical response and sweat chloride decrease, we had considered the possibility of insufficient blood concentration of ETI or its metabolites. Plasma level measurement were not available, and we attempted to verify indirectly this hypothesis by a combination of suppression of ETI metabolic pathway by posaconazole and dosages of ETI higher than usually administered in these circumstances. The patient was closely monitored, and no adverse events were recorded. Before starting posaconazole sweat chloride was 107 mEq/L and ppFEV1 36%, 7 days after sweat chloride was 100 mEq/L and ppFEV1 40%. After 3 months, in light of a further decrease in ppFEV1 (31%) and no clinical and sweat chloride level improvements (106 mEq/L), posaconazole was stopped and standard ETI dosage started again. Presently, the patient is under continuous antibiotic treatment, in stable conditions, (last ppFEV1 36%) and has been preliminarily evaluated at a Lung Transplant Unit.

TT190–clinical data

TT190 is a 21-years-old female, affected by Cystic Fibrosis diagnosed through neonatal screening and sweat test (125 mEq/l Cl-). A standard *CFTR* mutation panel identified F508del/E585X. Her history of CF is characterized by pancreatic insufficiency and severe lung disease with early pulmonary colonization by Methicillin-Sensitive *Staphylococcus aureus* (MSSA), *Stenotropomonas maltophilia* and from 2018 *Pseudomonas aeruginosa*.

Since 2015 she has been hospitalized annually for pulmonary exacerbations (PEx), and since 2018 she deteriorated further with more frequent hospital admissions (2-3/year) for PEx. In February 2019 she started non-invasive ventilation during night rest and exercise.

In January 2021 she was initiated on ETI, 2 standard dose tablets in the morning and one ivacaftor 150-mg tablet in the evening. At the time of treatment start she was under antibiotic treatment for PEx. After 10 days of treatment, her ppFEV1 showed no substantial changes, moving from 34 to 33. However, the patient reported clinical benefits in cough and dyspnea, and ETI was well tolerated. Two weeks after initiation, a significant ppFEV1 decrease (26) was observed, without any PEx clinical symptoms. A month later a clinical expressed PEx appeared, she was admitted to the CF Centre and at discharge her pp FEV1 was 40. In the following months she reported increasing cough, was started on continuative oral antibiotics and ppFEV1 decreased to 30 (Table x). She also had several episodes of moderate hemoptysis, a complication she had previously suffered from. Repeated post-ETI sweat tests showed high chloride levels with no major improvements: 94 mEq/L (February 2021), 102 mEq/L (March 2021), 105 mEq/L (July 2021), 97 mEq/L (November 2021) [Table S2]

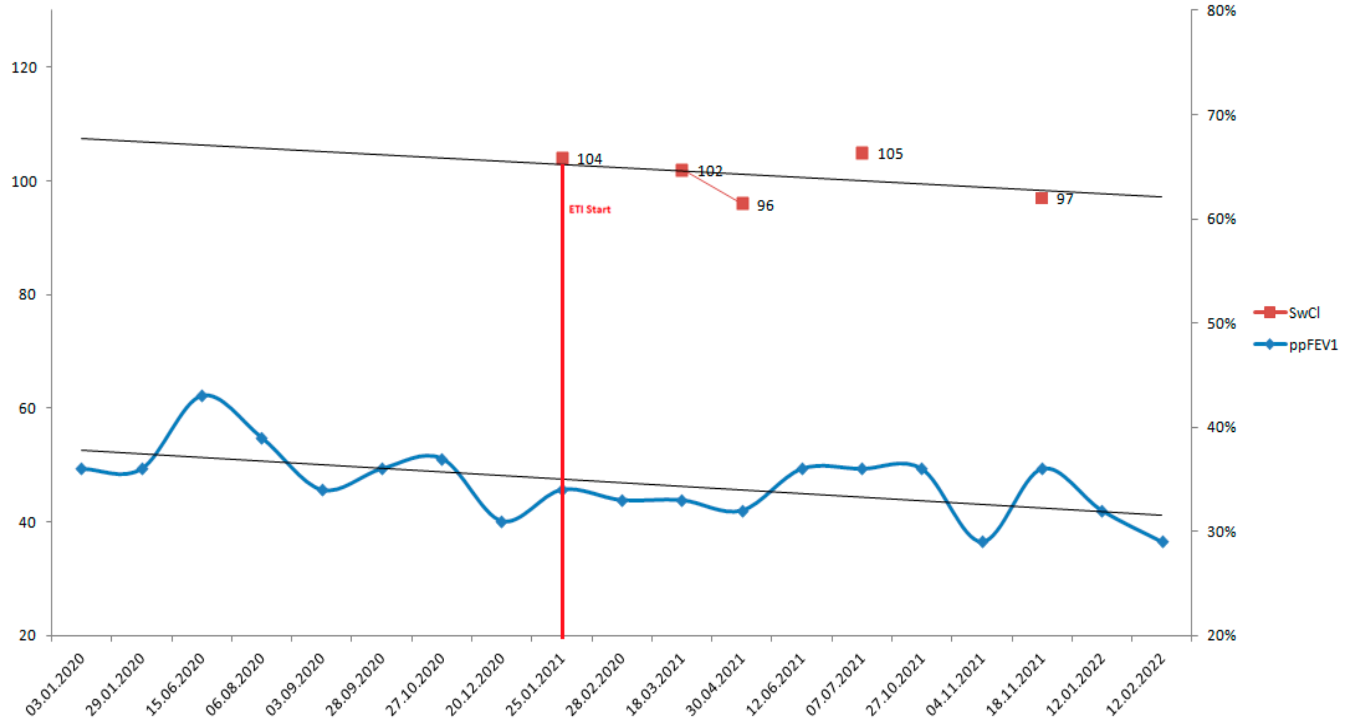

Table S2

TT003–clinical data

TT003 is a 34-years-old female, affected by Cystic Fibrosis diagnosed at one year of age because of symptoms and positive sweat test (Chloride 117 mEq/l). A standard *CFTR* mutation panel resulted in homozygosity for F508del. Her clinical history is characterized by pancreatic insufficiency and severe lung disease with early pulmonary colonization by Methicillin-Resistant *Staphylococcus aureus* (MRSA) and Multi-Drug Resistant *Pseudomonas aeruginosa*.

Since 2016 she has had frequent hospital admissions for PEx and recurrent pneumothorax episodes, and in 2018 has started continuous oxygen supplementation. Her ppFEV1 was stably under 40%, even after IV antibiotics, and in October 2017 she was enrolled in a waiting list for lung transplant.

In January 2020 she initiated ETI, 2 standard dose tablets in the morning and one ivacaftor 150-mg tablet in the evening. At the time of treatment start she had been under continuous IV antibiotic treatment for almost one year. After 3 days of treatment, her ppFEV1 showed a significant increase, moving from 24 to 33; the patient also reported

clinical benefits in cough and dyspnea. ETI was always well tolerated. In the following months we observed further clinical benefits, with no more need of oxygen supplementation or hospitalizations for PEx. Repeated post-ETI sweat tests showed significant improvements in chloride levels: 62 mEq/L (February 2020), 44 mEq/L (May 2020), 48 mEq/L (September 2020), 43 mEq/L (November 2020) [Table S3].

In November 2021 the patient was removed from lung transplant waiting list.

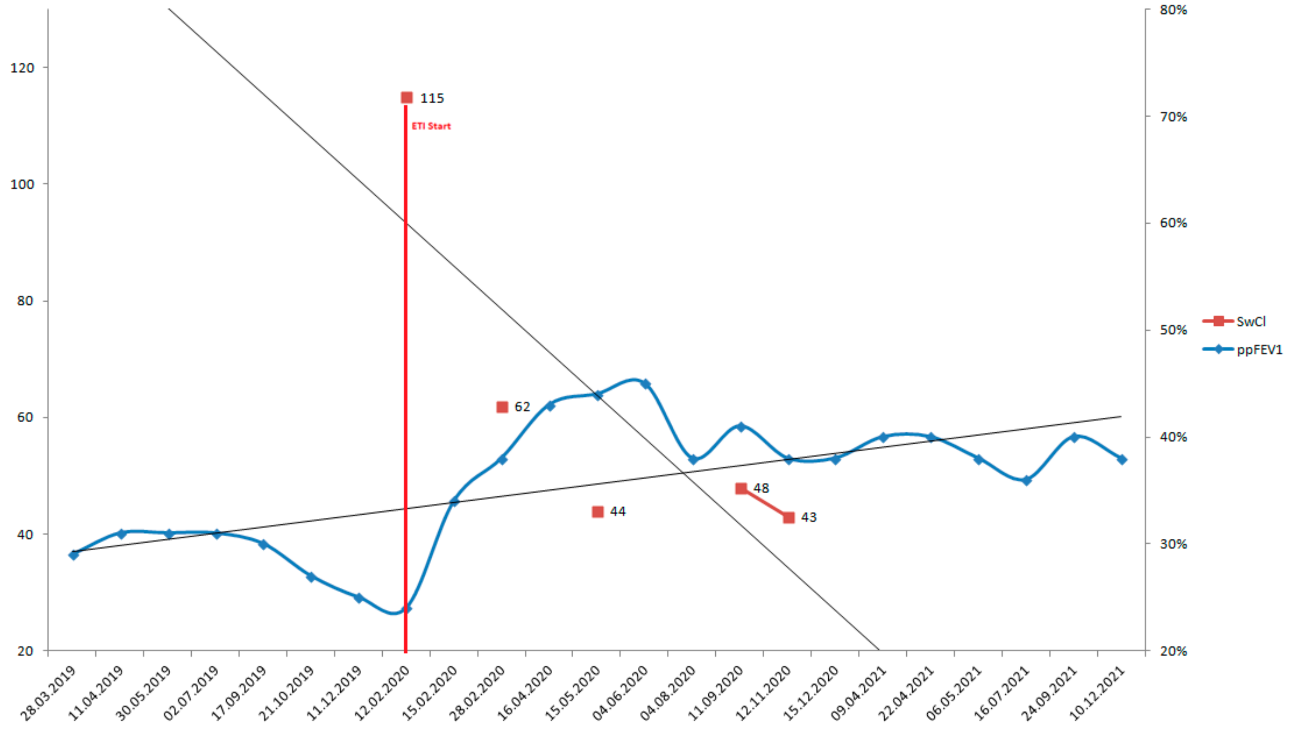

Table S3
